# Post-acute phase and sequelae management of epidermal necrolysis: an international, multidisciplinary DELPHI-based consensus

**DOI:** 10.1186/s13023-023-02631-7

**Published:** 2023-02-22

**Authors:** S. Ingen-Housz-Oro, V. Schmidt, M. M. Ameri, R. Abe, A. Brassard, A. Mostaghimi, A. S. Paller, A. Romano, B. Didona, B. H. Kaffenberger, B. Ben Said, B. Y. H. Thong, B. Ramsay, E. Brezinova, B. Milpied, C. G. Mortz, C. Y. Chu, C. Sotozono, J. Gueudry, D. G. Fortune, S. M. Dridi, D. Tartar, G. Do-Pham, E. Gabison, E. J. Phillips, F. Lewis, C. Salavastru, B. Horvath, J. Dart, J. Setterfield, J. Newman, J. T. Schulz, A. Delcampe, K. Brockow, L. Seminario-Vidal, L. Jörg, M. P. Watson, M. Gonçalo, M. Lucas, M. Torres, M. H. Noe, N. Hama, N. H. Shear, P. O’Reilly, P. Wolkenstein, P. Romanelli, R. P. Dodiuk-Gad, R. G. Micheletti, G. S. Tiplica, R. Sheridan, S. Rauz, S. Ahmad, S. L. Chua, T. H. Flynn, W. Pichler, S. T. Le, E. Maverakis, S. Walsh, L. E. French, M. C. Brüggen

**Affiliations:** 1 Department of Dermatology, AP-HP, Henri Mondor Hospital, 1 Rue Gustave Eiffel, 94000 Créteil, France; 2 ToxiTEN Group, European Reference Network for Rare Skin Diseases, Paris, France; 3 Reference Center for Toxic Bullous Dermatoses and Severe Drug Reactions TOXIBUL, Créteil, France; 4 EpiDermE, Université Paris Est Créteil, Créteil, France; 5 University Hospital Basel, Basel, Switzerland; 6 Faculty of Medicine, University of Zurich, Zurich, Switzerland; 7 Department of Dermatology, University Hospital Zurich, Zurich, Switzerland; 8 Christine Kühne-Center for Allergy Research and Education, Davos, Switzerland; 9 Division of Dermatology, Niigata University Graduate School of Medical and Dental Sciences, Niigata, Japan; 10 Department of Dermatology, UC Davis Medical Center, Sacramento, CA USA; 11 Department of Dermatology, Brigham and Women’s Hospital, Boston, MA USA; 12 Department of Dermatology, Northwestern University Feinberg School of Medicine, Chicago, IL USA; 13 Oasi Research Institute-IRCCS, Troina, Italy; 14 Rare Disease Unit, I Dermatology Division, Istituto Dermopatico Dell’Immacolata, IRCCS, Rome, Italy; 15 The Ohio State University Wexner Medical Center Division of Dermatology, Upper Arlington, OH USA; 16 Department of Dermatology, CHU Edouard Herriot, Lyon, France; 17 Department of Rheumatology, Allergy and Immunology, Tan Tock Seng Hospital, Singapore, Singapore; 18 Department of Dermatology, University Hospital Limerick, Limerick, Ireland; 19 First Department of Dermatovenereology, Masaryk University Faculty of Medicine, St. Ann’s Faculty Hospital in Brno, Brno, Czech Republic; 20 Department of Adult and Pediatric Dermatology, Bordeaux University Hospitals, Bordeaux, France; 21 Department of Dermatology and Allergy Center, Odense Research Center for Anaphylaxis (ORCA), Odense University Hospital, Odense, Denmark; 22 Department of Dermatology, National Taiwan University Hospital, National Taiwan University College of Medicine, No. 7, Chung-Shan South Road, Taipei, 10002 Taiwan; 23 Department of Ophthalmology, Kyoto Prefectural University of Medicine, 465 Kajii-Cho, Hirokoji-Agaru, Kawaramach-Dori, Kamigyo-Ku, Kyoto, 602-0841 Japan; 24 Department of Ophthalmology, CHU Charles-Nicolle, Rouen, France; 25 Department of Psychology, University of Limerick, Limerick, Ireland; 26 MICORALIS Laboratory, Department of Periodontology, Faculty of Dentistry, Côte d’Azur University, Saint Roch Hospital, Nice, France; 27 Department of Dermatology, University of California Davis, Sacramento, CA USA; 28 Department of Internal Medicine, Centre Hospitalier Intercommunal de Créteil, Créteil, France; 29 Fondation Ophtalmologique Adolphe de Rothschild, Paris, France; 30 Institute for Immunology and Infectious Diseases, Murdoch University, Murdoch, WA Australia; 31 St John’s Institute of Dermatology, Guy’s and St Thomas’ Hospital, London, UK; 32 Department of Paediatric Dermatology, Colentina Clinical Hospital, Carol Davila University of Medicine and Pharmacy, Bucharest, Romania; 33 Department of Dermatology, University Medical Center Groningen, University of Groningen, Hanzeplein 1, 9700 RB Groningen, The Netherlands; 34 Moorfields Eye Hospital NHS Foundation Trust, The UCL Institute of Ophthalmology, London, UK; 35 Department of Oral Medicine, Guy’s and St Thomas’ NHS Foundation Trust, London, UK; 36 Department of Dermatology, King’s College Hospital NHS Foundation Trust, London, UK; 37 Division of Burns, Massachusetts General Hospital, Boston, 02114 USA; 38 Department of Ophthalmology, CHU Bichat-Claude Bernard, Paris, France; 39 Department of Dermatology and Allergy Biederstein, School of Medicine, Technical University of Munich, Munich, Germany; 40 Department of Dermatology and Cutaneous Surgery, University of South Florida, Tampa, FL USA; 41 Division of Allergology and Clinical Immunology, Department of Pneumology, Inselspital, Bern University Hospital, University of Bern, Bern, Switzerland; 42 Cornea and External Eye Disease Service, Moorfields Eye Hospital, London, UK; 43 Department of Dermatology, Coimbra University Hospital Center, Faculty of Medicine, University of Coimbra, Coimbra, Portugal; 44 Medical School, University of Western Australia, Perth, WA 6009 Australia; 45 Allergy Unit, IBIMA-Regional University Hospital of Malaga-UMA, Málaga, Spain; 46 Department of Dermatology, Brigham and Women’s Hospital, Harvard Medical School, Boston, MA USA; 47 Department of Dermatology, University of Toronto, Toronto, ON Canada; 48 Department of Nursing and Midwifery, University of Limerick, Limerick, Ireland; 49 Dr. Phillip Frost Department of Dermatology and Cutaneous Surgery, University of Miami, Miller School of Medicine, Miami, FL USA; 50 Dermatology Department, Emek Medical Center, Bruce Rappaport Faculty of Medicine, Technion - Institute of Technology, Haifa, Israel; 51 Department of Dermatology and Medicine, Perelman School of Medicine, University of Pennsylvania, Philadelphia, PA USA; 52 2Nd Department of Dermatology, Colentina Clinical Hospital, Carol Davila University of Medicine and Pharmacy, Bucharest, Romania; 53 Burn Service, Boston Shriners Hospital for Children, Boston, MA USA; 54 Academic Unit of Ophthalmology, Birmingham and Midland Eye Centre, Institute of Inflammation and Ageing, University of Birmingham, Birmingham, UK; 55 Department of Dermatology, University Hospitals Birmingham NHS Foundation Trust, Birmingham, UK; 56 Ophthalmology, Bon Secours Hospital, Cork, Ireland; 57 ADR-AC GmbH, Bern, Switzerland; 58 Department of Dermatology, University Hospital, Munich University of Ludwig Maximilian, Munich, Germany; 59 Dr Phillip Frost Department of Dermatology and Cutaneous Surgery, University of Miami Miller School of Medicine, Miami, FL USA; 60 Department of Medicine, Vanderbilt University Medical Center, Nashville, TN USA; 61 Department of Immunology, Sir Charles Gairdner Hospital, Pathwest Laboratory Medicine, Perth, WA 6009 Australia; 62 Sunnybrook Health Sciences Centre, Toronto, ON Canada; 63 Division of Burns, Massachusetts General Hospital, Boston, MA USA; 64 Department of Surgery, Harvard Medical School, Boston, MA USA; 65 Department of Medicine, University of Toronto, Toronto, Canada

**Keywords:** Epidermal necrolysis, Stevens-Johnson syndrome, Toxic epidermal necrolysis, Sequelae, Quality of life, Delphi, Consensus

## Abstract

**Background:**

Long-term sequelae are frequent and often disabling after epidermal necrolysis (Stevens-Johnson syndrome (SJS) and toxic epidermal necrolysis (TEN)). However, consensus on the modalities of management of these sequelae is lacking.

**Objectives:**

We conducted an international multicentric DELPHI exercise to establish a multidisciplinary expert consensus to standardize recommendations regarding management of SJS/TEN sequelae.

**Methods:**

Participants were sent a survey via the online tool “Survey Monkey” consisting of 54 statements organized into 8 topics: general recommendations, professionals involved, skin, oral mucosa and teeth, eyes, genital area, mental health, and allergy workup. Participants evaluated the level of appropriateness of each statement on a scale of 1 (extremely inappropriate) to 9 (extremely appropriate). Results were analyzed according to the RAND/UCLA Appropriateness Method.

**Results:**

Fifty-two healthcare professionals participated. After the first round, a consensus was obtained for 100% of 54 initially proposed statements (disagreement index < 1). Among them, 50 statements were agreed upon as ‘appropriate’; four statements were considered ‘uncertain’, and ultimately finally discarded.

**Conclusions:**

Our DELPHI-based expert consensus should help guide physicians in conducting a prolonged multidisciplinary follow-up of sequelae in SJS-TEN.

**Supplementary Information:**

The online version contains supplementary material available at 10.1186/s13023-023-02631-7.

## Introduction

Epidermal necrolysis (EN), including Stevens-Johnson syndrome (SJS) and toxic epidermal necrolysis (TEN, or Lyell syndrome) is a rare but severe delayed hypersensitivity reaction characterized clinically by purpuric macules, a variable extent of epidermal detachment (SJS, < 10% detached-detachable body surface area, overlap syndrome 10–29%, TEN, ≥ 30%) and mucous membrane (MM) involvement [1]. EN is induced by drugs in 85% of cases, but some cases remain “idiopathic” [2, 3]. Pathophysiology leading to epidermis and epithelia apoptosis and necrolysis is complex [4, 5]. The incidence ranges from 1–2 to 6 cases per million inhabitants per year [6]. Overall mortality, which can be predicted on an individual scale by the SCORTEN in the acute phase, is approximately 15% [7–9]. The cornerstones of management during the acute phase are the quick cessation of the culprit drug, and optimized supportive care in reference centers [10, 11]. International guidelines, including a recent DELPHI exercise, summarized the key points of this management [12–15].

After the acute phase, long-term sequelae have been described, with significant impact on the quality of life. The main ones are cutaneous (e.g. pigmentation issues, hypertrophic scars), ocular (from minor dryness to severe conjunctival inflammation, synechiae and corneal defects that may lead to blindness), and psychological distress [16–21]. Consequently, a prolonged multidisciplinary follow-up is warranted, but there is no consensus about the practical modalities of this follow-up.

Our aim in this multicenter DELPHI exercise was to harmonize modalities of follow-up of patients after the acute phase of EN.

## Methods

### Panel selection

The project was initiated by the SJS/TEN subgroup (ToxiTEN group) of the skin European Reference Network (ERN-skin), which is composed of dermatologists. An international multidisciplinary panel of experts in the field of EN was subsequently established. Participants were identified from academic centers that provide inpatient dermatology or intensive care services specialized in EN patient care. In total, 77 experts were identified and invited via email to participate in the DELPHI exercise. Of them, there were 39 dermatologists, 14 allergologists, 12 ophthalmologists, 3 psychologists/psychiatrists, 3 gynecologists, 3 nurses specifically involved in EN, 2 burn surgeons and 1 dentist. All participants were solicited to assess only the statements in their field of expertise.

**Fig. 1:**
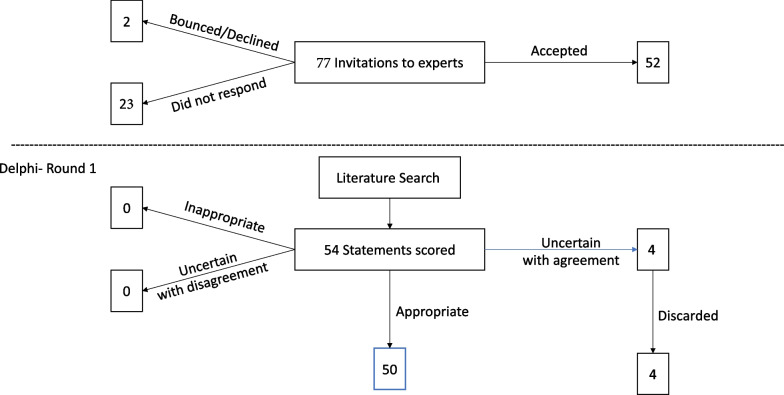
Top: Flow chart of the experts. Bottom: Flow chart of the statements

Of the 77 identified experts, 23 did not respond to the invitation to participate, 2 declined, and the remaining 52 agreed to participate (Fig. 1).

### Statements

Participants were sent an online survey consisting of 54 statements regarding EN after-care follow-up. Statements were established by the steering committee of the study (four experts of epidermal necrolysis [MCB, LF, SW, SIHO], and one dermatology resident [VS]). The statements were based on previously published studies and reviews on the topic as well as our routine practice experience and assessment of SJS/TEN patient management [13]. We screened Pubmed for literature on SJS/TEN aftercare in the past 15 years (2006–2021, papers in English; search keywords: toxic epidermal necrolysis, Stevens-Johnson syndrome, AND sequelae, quality of life, follow-up). Different types of articles were included: general/review articles about EN  and original articles on the main physical (eyes, skin, genital, dental) and psychological sequelae and health-related quality of life in this disease. For the topic of allergological work-up, we screened Pubmed literature without time limitation using the following key words: toxic epidermal necrolysis, Stevens-Johnson syndrome, AND allergological work-up, skin testing, in vitro tests, patch tests, lymphocyte transformation test. Statements were organized into 8 categories: general recommendations, professionals involved, skin, oral mucosa and teeth, eyes, genital area, mental health, and allergy workup (in vivo and in vitro tests).

An online tool, “Survey Monkey”, was used to distribute surveys. Participants were asked to evaluate the level of appropriateness of statements on a scale of 1 (extremely inappropriate) to 9 (extremely appropriate). Participants were given the option of selecting “N/A” (not applicable) if they felt they did not have the necessary expertise to rank a particular statement. Participants also had the opportunity to submit comments to be incorporated into subsequent DELPHI rounds. Members of the steering committee (MCB, LF, SW, SIHO) did not respond to the survey.

### Statistics

Results were analyzed according to the RAND/UCLA Appropriateness Method. The median rating for appropriateness, interpercentile range (IPR), interpercentile range adjusted for symmetry (IPRAS), and disagreement index (DI) were calculated (DI = IPR/IPRAS) for each statement [22]. Median appropriateness values were assessed as follows: 1.0 to 3.4 was considered “inappropriate”, 3.5 to 6.9 as “uncertain” and 7.0 to 9.0 as “appropriate.” A disagreement index (DI) < 1 indicated a consensus obtained among the participants in terms of a statements’ appropriateness.

## Results

### Participants and DELPHI exercise

Fifty-two of the 77 participants (Fig. 1 Top) who agreed to participate in the DELPHI exercise responded (67.5% response rate). Participants (women n = 26) were from 8 specialties (dermatologists n = 24, allergologists n = 11, participants from 6 other specialties n = 17), and 18 different countries on 4 continents. There were no significant differences in terms of sex and specialties among responders and non-responders (data not shown).

The statements on which the panel ‘agreed’ were ‘appropriate’ were used for the consensus.

### First round

A consensus was reached for all of the 54 statements (100%). Four statements were labelled as ‘uncertain’ in the section “Eyes“, “Mental health“ and “Allergological workup“. Statements were discarded based on the discussion and reassessment by the steering committee (Fig. 1 Bottom). The statements that were discarded addressed the (non-)recommendation of corneal transplantation, additional measures such as hypnosis to reduce symptoms of anxiety or depression and the type and timing of allergological testing after re-epithelialization.

All statements with the respective DIs and medians are displayed in Table 1. All statements were agreed upon, with a DI < 1 (i.e. reached the necessary level of agreement). IPR and IPRAS values are displayed in Additional file 1: Table S1.

**Table 1:** Items of the DELPHI-based exercise for the management of sequelae in epidermal necrolysis

	Disagreement index (DI)*	Median
Items the panel agreed were ‘appropriate’		
General recommendations		
A follow-up control SHOULD be performed 1–2 months after discharge from the hospital and regularly thereafter as needed	0	9
Professionals involved		
Patients SHOULD be managed by a multidisciplinary team	0	9
The DERMATOLOGIST SHOULD lead in the management of follow-up	0.531	8
An OPHTHALMOLOGIST SHOULD be involved in case of ocular involvement	0	9
Support by a PSYCHIATRIST and/or PSYCHOLOGIST SHOULD be offered	0.132	9
A DENTIST and/or a STOMATOLOGIST SHOULD be involved in case of chronic oral mucosal involvement	0.132	9
An ENT specialist SHOULD be involved after discharge if there was nasopharyngeal and/or laryngeal involvement in the acute phase	0.132	9
A UROLOGIST SHOULD be involved in cases of severe genital involvement, where a risk of urethral synechiae/strictures exists	0	9
A GYNECOLOGIST SHOULD be involved in case of severe genital involvement, where a risk of vaginal synechiae/strictures exists	0	9
A PULMONOLOGIST SHOULD be involved after discharge if there was pulmonary involvement in the acute phase	0.132	9
A SOCIAL WORKER SHOULD be involved if needed	0	9
A DIETICIAN SHOULD be involved if needed	0.292	9
Skin		
Patients SHOULD practise careful sun protection post-discharge	0	9
Patients SHOULD apply emollients daily	0.262	9
Laser treatment MAY be considered for hypertrophic scars	0.374	7
Residual skin pain SHOULD be further investigated	0.292	8
A NEUROLOGIST or a PAIN SPECIALIST SHOULD be involved in patients with chronic skin pain	0.132	9
Oral mucosa and teeth		
Patients SHOULD receive specific instructions for dental health	0.262	9
Patients who had oral mucosa involvement SHOULD have regular dental check-ups	0.132	9
Specific therapy SHOULD be implemented in patients with xerostomia	0.019	9
Saliva substitutes SHOULD be used in patients with xerostomia	0.132	9
Topical sialagogues MAY be considered in patients with xerostomia	0.292	8
Eyes		
Patients SHOULD undergo a complete ophthalmological examination as often as needed	0	9
An OPHTHALMOLOGIST SHOULD guide the medical treatment of ocular symptoms	0	9
A combination of artificial tears without preservatives and topical vitamin A SHOULD be used in patients with xerophthalmia	0.319	8
The use of topical cyclosporine or other immunosuppressive agent MAY be proposed in patients with severe xerophthalmia	0.374	7
The use of scleral lenses SHOULD be considered in patients with severe xerophthalmia and/or scarring	0.292	8
Surgical ocular surface reconstruction SHOULD be considered as a last resort in patients with extensive scarring	0.724	7
Genital area		
Sequelae such as vulvodynia, vulvar and vaginal synechiae SHOULD be assessed after epithelialization	0.018	9
Topical corticosteroids SHOULD be considered in patients with vulvar and/or vaginal synechiae to reduce extensive scarring	0.292	8
Surgical correction SHOULD be considered in cases of extensive vulvar and/or vaginal scarring	0.132	9
Emollients SHOULD be used to avoid vulvar and vaginal dryness	0.132	9
Mental health		
Every follow-up control SHOULD include a screening for psychological well-being	0.132	9
This screening SHOULD include questions on the quality of sleep, mood status, anxiety, nightmares, and symptoms of depression	0	9
A standardized tool such as hospital anxiety and depression score (HADS) MAY be helpful in the screening for psychological well-being	0.132	9
Psychological support SHOULD be actively offered to patients with chronic disabling sequelae	0	9
A psychological and/or psychiatric follow-up CAN help to reduce issues like post-traumatic stress disorder	0	9
Iatrogenic psychiatric symptoms SHOULD be excluded	0.292	8
Psychotropic drugs MAY be considered according to the psychiatrist’s evaluation	0.292	9
Allergy workup		
A preliminary allergy card prohibiting the use of ALL suspect drugs MUST be given to the patient upon release from the hospital	0	9
The patient MUST be clearly informed during the hospital stay about the suspect drug(s), their avoidance and cross-reactivity	0	9
The patient’s companion/family MUST be clearly informed during the hospital stay about the suspect drug(s), their avoidance and cross-reactivity	0	9
Prick tests SHOULD NOT be routinely performed	0.132	9
Intradermal tests SHOULD NOT be routinely performed	0.292	9
If available, a lymphocytic transformation test (LTT) CAN be useful in the diagnostic work-up	0.492	8
If available, an Elispot test CAN be useful in the diagnostic work-up	0.748	7
A drug CANNOT be excluded as culprit agent solely based on negative results of any of the allergological tests	0	9
A definitive allergy card MUST be given to the patient after the allergy work-up	0	9
The patient MUST be clearly informed about the drug(s) on the allergy card, their avoidance and cross-reactivity after the allergy work-up	0	9
The general practitioner and all physicians involved in the management of the patient MUST be informed about the drug(s) on the allergy card, their avoidance and cross-reactivity after the allergy work-up	0	9
Items the panel agreed were ‘uncertain’		
Eyes		
Corneal transplantation SHOULD NOT be recommended due to the risk of clinical exacerbation	0.652	5
Mental health		
Additional measures such as hypnosis MAY help reducing symptoms of anxiety or depression	0.652	5
Allergy workup		
Allergological testing SHOULD be performed at least 6–8 weeks after complete re-epithelization	0.652	6
Patch-tests SHOULD be performed for the diagnostic work-up	0.519	5
Items the panel agreed were ‘inappropriate’		
None		
Items the panel disagreed		
None		

^*^A disagreement index value greater than 1 indicates a lack of consensus; below 1 indicates a consensus

## Discussion

Thanks to this DELPHI exercise, we obtained for the first time an international consensus for the main objectives of an optimal and standardized multidisciplinary follow-up of patients after the acute phase of SJS/TEN. Based on previous literature, we questioned multidisciplinary experts with the RAND/UCLA appropriateness method, developed by RAND corporation with clinicians at the University of California at Los Angeles (UCLA) and widely used for DELPHI exercises [22], and reached formal agreement for the cornerstones of the patients’ long-term follow-up.

Consensus was obtained after one round in key fields of patient management: general recommendations, professionals involved, follow-up care for skin, oral mucosa and teeth, eyes, genital area, and mental health and allergological workup. Only 4 statements remained ‘uncertain’. All other 50 statements resulted in an ‘appropriate’ consensus.

Regular follow-up within the first year after the acute phase must be organized and driven by a dermatologist, then after, tailored accordingly to the needs of each patient. The vast spectrum of potential sequelae requires a collaborating panel of medical specialists organized by the dermatologist for optimal care: ophthalmologist, psychiatrist/psychologist, ENT specialist, gynecologist/urologist, odontologist/dentist, pulmonologist, neurologist, and others according to the patient’s need. Furthermore, the intervention of a dietician may be mandatory within initial few weeks or months due to the frequent loss of weight during the acute phase. If required, based on the patient’s social situation and the local setup, a social worker may facilitate rehabilitation through helping to obtain financial support, especially in case of disability (e.g. visual impairment), and sometimes financial compensation for medical (iatrogenic) accident [23].

Skin sequelae are very frequent and include pigmentation disorders, hypertrophic scars, polymorphic nail changes and occasionally chronic skin pain [16, 17, 24, 25]. All of these issues can have a major impact on the patient’s quality of life. Consensus was obtained for prolonged sun protection and daily use of emollients. However, it is not possible to provide guidelines for the duration of these measures, which should be individualised for each patient, depending on his/her phototype and quality of healing. Consensus was also obtained for a consideration of laser procedures for disabling hypertrophic scars, and involvement of a neurologist in the exploration and management of chronic pain. The latter was shown to result from sensitization of both small-diameter (burning and itching sensations) and large-diameter nerve fibers (allodynia), and major affective and emotional components [26]. In our study, we did not provide statements about nail sequelae. The latter are frequent and polymorphic and often disabling for the patient, but best management guidelines are still missing [24].

Dental sequelae and xerostomia are frequent [27]. Consensus was obtained for a regular dental follow-up, for the necessary duration for each individual patient after the acute phase, and prescription of saliva substitutes or systemic or topical sialogogues such as pilocarpine or cevimeline, as have been described as effective in other xerostomia and hyposalivation syndromes [28].

Ocular sequelae are the main disabling sequela. Ocular changes of variable severity affect up to 75% of survivors with an impact on daily personal and professional life. Risk factors are the severity of the disease at the acute phase, including the severity of ocular involvement [18, 29]. Ocular change include dryness, ectropion, entropion, trichiasis, meibomian gland dysfunction, corneal erosions, ulcerations, neovascularization, stromal scarring, and conjunctivalization of the corneal surface with loss of visual acuity [16]. Consensus was obtained for the regular use of artificial tears without preservatives and topical vitamin A in xerophthalmia, together with a trial of an adjunctive topical immunosuppressant such as cyclosporine, the latter was demonstrated to be efficacious in a small study in EN as well as in other causes of dry eye, although may be poorly tolerated (pain, redness, and eyelid swelling) [30, 31]. Topical tacrolimus is an alternative, with interesting results on ocular surface persistent inflammation [32]. Scleral lens or tear-exchangeable, limbal, rigid contact lenses are used in the most disabling ocular surface sequelae, with good results in term of dryness improvement, visual rehabilitation, and global ocular comfort [33–35]. In contrast, the statement that corneal transplantation should not be recommended due to the risk of clinical exacerbation led to an ‘uncertain’ consensus result. Few studies were published on this topic, but outcome of keratoplasty in cicatrizing conjunctival diseases is poor, causing a further deterioration of vision and morbidity due to persistence of epithelial defects, stromal ulceration, perforation, and graft rejection [36].

In women, genital sequelae are not uncommon (about 20%) and include labial agglutination, introital stenosis, vaginal synechiae and stenosis, vaginal and vulval adenosis, hematocolpos, and hematometra, leading to dryness, dyspareunia and bleeding [37]. Although genitourinary sequelae are rare in children, caution is needed in this population [38, 39]. In our DELPHI exercise, consensus was obtained for the use of emollients to reduce vulvar or vaginal dryness, topical corticosteroids in case of synechiae to reduce the risk of more extensive scarring, and surgical correction in case of extensive synechiae, which require trained surgeons [40].

Psychological sequelae are frequent. Medicine avoidance is common, given that the disease is drug induced in the majority of cases. Other conditions include anxiety, depression, and nightmares. Of note, within the 6 months after the acute phase, 25% of patients develop post-traumatic stress disorder, especially in case of previous psychological fragility [19, 20]. Consensus was obtained for screening for psychological well-being at each follow-up appointment. This screening may be first conducted by the dermatologist, using standardized tools, such as the hospital anxiety and depression scale (HADS). HADS is reliable and easy to use self-assessment instrument developed 40 years ago for detecting states of depression (7 questions) and anxiety (7 questions) to guide the need for specialized intervention [41]. Intervention involves care from both a psychologist and a psychiatrist to prescribe psychotropic drugs according to each patient’s needs. Additional measures such as hypnosis to reduce symptoms of anxiety or depression remained of ‘uncertain’ appropriateness. Indeed, whereas the efficacy of hypnosis and other techniques such as relaxation was suggested in burns [42, 43], literature still lacks in psychological sequelae of EN.

Pulmonary sequelae are rare. A pulmonologist should be involved in case of severe initial lung involvement. The most frequent sequela is diffusion impairment, most often asymptomatic and this may be screened for by pulmonary function tests [44]. Other sequelae, especially bronchiolitis obliterans, are very rare and mostly described in children [45]. Due to this rarity, no statement was proposed to experts about pulmonary sequelae.

Prevention of relapse of EN is essential. Consensus was obtained for provision of an allergy card to the patient from the hospital discharge, giving clear information about the culprit drug(s) and all related contraindications (drugs of the same biochemical family / cross-reactivity). The patients, their family, the pharmacist and the primary care provider must be clearly informed of the suspected drugs, the need for avoidance and the drugs that may cross-react with the suspected agent(s).

Allergy workup may help for both the identification of the culprit drug(s) and identification of an alternative medication that may be used in the future. Performing the allergy work-up at a suggested time point of 6–8 weeks was rated as “uncertain” by the group. This statement was initially designed for cutaneous tests, but was maybe not precise enough, and it is possible that some experts understood the phrasing for in vitro tests also. Given the lack of precise literature on the topic, we decided to not suggest an alternative statement. The best time to perform tests remains to be further studied. Patch-tests to explore EN have been described in several small series, showing a positivity rate lower than in other drug reactions, but varied upon the drug tested (9 to 62%) [46–48]. Surprisingly, the statement that patch-tests should be performed for the diagnostic work-up remained ‘uncertain’ among experts. This is probably explained by the low rate of positivity, especially for high-risk drugs such as sulfasalazine and allopurinol which give always negative results [49], and by the fact that some teams would prefer performing in vitro tests very soon after the onset of the reaction [50]. To date, data about the usefulness and the safety of prick and intradermal tests are too scarce to recommend their use in EN. Consequently, to date, only patch-tests, which are safe in EN, are recommended in guidelines [47, 51, 52]. Even in case of negative results, provocation tests with the most highly suspected culprit drug(s) must not be performed in EN patients [53]. Lymphocyte transformation test, eventually combined with cytokine detection assays, may be useful if performed shortly after the acute phase, showing a positivity rate correlated to the ALDEN [50, 54, 55]. Other in vitro tests such as IFN-γ release Enzyme-Linked Immunosorbent Spot (ELISpot) assay may be useful, combined with in vivo skin testing [56]. However, these in vitro tests are often not available routinely available, and large series to assess their usefulness in EN are lacking. After allergy workup, a definitive allergy card must be given to the patients, with all information about the drug(s) contraindicated for the rest of their life and the list of cross-reactivities.

## Conclusion

SJS and TEN are delayed-type severe hypersensitivity reactions associated with a high risk of long-term mucocutaneous disabling sequelae. Here, through a multidisciplinary consensus based on a DELPHI exercise, we propose for the first time a harmonization of practices emphasizing on the key points of a multidisciplinary long-term follow-up. The best timing of follow-up visits and the duration of the latter considerably vary among patients and depend on the severity of sequelae. The current expert consensus provides general recommendations that need to be case-to-case adapted (Table 2) and highlights the need to further determine details of follow-up care and to investigate the best management for rare but disabling sequelae in SJS/TEN patients.

**Table 2:** Proposition for the follow-up multidisciplinary calendar after the acute phase

1–2 months after discharge	Dermatologist (skin sequelae and coordination of the follow-up) Ophthalmologist Psychiatrist and/or psychologist ENT Pneumologist if needed (± pulmonary function tests) Gynecologist/urologist if needed (scarring) Dietetician if needed (loss of weight) Social worker if needed
6 months* after the acute phase	Same specialties according the needs of the patient Stomatologist/dentist Allergy work-up
1 year* after the acute phase	Same specialties according the needs of the patient Prolonged follow-up by the dermatologist to screen the needs of the patient and coordinate the multidisciplinary follow-up

^*^Timing recommendations not part of the current DELPHI consensus

## Supplementary Information

**Additional file 1**.** Table S1**. Disagreement index (DI), interpercentile range (IPR) and interpercentile range adjusted for symmetry (IPRAS) for all statements.

## Data Availability

The dataset(s) supporting the conclusions of this article is(are) included within the article (and its additional file(s)).

## Abbreviations

EN: Epidermal necrolysis
SJS: Stevens-Johnson syndrome
TEN: Toxic epidermal necrolysis
MM: Mucous membranes
ERN: European Reference Network
IPR: Interpercentile range
IPRAS: Interpercentile range adjusted for symmetry
DI: Disagreement index
ENT: Ear nose throat
ALDEN: Algorithm of drug causality for epidermal necrolysis
