# Supplementary material for: Post-acute phase and sequelae management of epidermal necrolysis: an international, multidisciplinary DELPHI-based consensus
Source: Orphanet J Rare Dis. 2023 Feb 22;18:33. doi: 10.1186/s13023-023-02631-7 (PMC9945700; doi:10.1186/s13023-023-02631-7)
Supplement: Supplementary file 1 — Additional file 1. Table S1. Disagreement index (DI), interpercentile range (IPR) and interpercentile range adjusted for symmetry (IPRAS) for all statements. [file 13023_2023_2631_MOESM1_ESM.docx]

**Table S1. Disagreement index (DI), interpercentile range (IPR) and interpercentile range adjusted for symmetry (IPRAS) for all statements**

|  | | | Disagreement index (DI)* | Interpercentile range (IPR) | Interpercentile range adjusted for symmetry (IPRAS) |
| --- | --- | --- | --- | --- | --- |
| General recommendations | | |  |  |  |
| A follow-up control SHOULD be performed 1 to 2 months after discharge from the hospital and regularly thereafter as needed. | | | 0 | 0 | 8.35 |
| Professionals involved | | |  |  |  |
| Patients SHOULD be managed by a multidisciplinary team. | | | 0 | 0 | 8.35 |
| The DERMATOLOGIST SHOULD lead in the management of follow-up. | | | 0.531 | 3.17 | 5.973 |
| An OPHTHALMOLOGIST SHOULD be involved in case of ocular involvement. | | | 0 | 0 | 8.35 |
| Support by a PSYCHIATRIST and/or PSYCHOLOGIST SHOULD be offered. | | | 0.132 | 1 | 7.6 |
| A DENTIST and/or a STOMATOLOGIST SHOULD be involved in case of chronic oral mucosal involvement. | | | 0.132 | 1 | 7.6 |
| An ENT specialist SHOULD be involved after discharge if there was nasopharyngeal and/or laryngeal involvement in the acute phase. | | | 0.132 | 1 | 7.6 |
| A UROLOGIST SHOULD be involved in cases of severe genital involvement, where a risk of urethral synechiae/strictures exists. | | | 0 | 0 | 8.35 |
| A GYNECOLOGIST SHOULD be involved in case of severe genital involvement, where a risk of vaginal synechiae/strictures exists. | | | 0 | 0 | 8.35 |
| A PULMONOLOGIST SHOULD be involved after discharge if there was pulmonary involvement in the acute phase. | | | 0.132 | 1 | 7.6 |
| A SOCIAL WORKER SHOULD be involved if needed. | | | 0 | 0 | 8.35 |
| A DIETICIAN SHOULD be involved if needed. | | | 0.292 | 2 | 6.85 |
| Skin | | |  |  |  |
| Patients SHOULD practise careful sun protection post-discharge. | | | 0 | 0 | 8.35 |
| Patients SHOULD apply emollients daily. | | | 0.262 | 1.83 | 6.978 |
| Laser treatment MAY be considered for hypertrophic scars. | | | 0.374 | 2 | 5.35 |
| Residual skin pain SHOULD be further investigated. | | | 0.292 | 2 | 6.85 |
| A NEUROLOGIST or a PAIN SPECIALIST SHOULD be involved in patients with chronic skin pain. | | | 0.132 | 1 | 7.6 |
| Oral mucosa and teeth | | |  |  |  |
| Patients SHOULD receive specific instructions for dental health. | | | 0.262 | 1.83 | 6.978 |
| Patients who had oral mucosa involvement SHOULD have regular dental check-ups. | | | 0.132 | 1 | 7.6 |
| Specific therapy SHOULD be implemented in patients with xerostomia. | | | 0.019 | 0.16 | 8.23 |
| Saliva substitutes SHOULD be used in patients with xerostomia. | | | 0.132 | 1 | 7.6 |
| Topical sialagogues MAY be considered in patients with xerostomia. | | | 0.292 | 2 | 6.85 |
| Eyes | | |  |  |  |
| Patients SHOULD undergo a complete ophthalmological examination as often as needed. | | | 0 | 0 | 8.35 |
| An OPHTHALMOLOGIST SHOULD guide the medical treatment of ocular symptoms. | | | 0 | 0 | 8.35 |
| A combination of artificial tears without preservatives and topical vitamin A SHOULD be used in patients with xerophthalmia. | | | 0.319 | 2.15 | 6.738 |
| The use of topical cyclosporine or other immunosuppressive agent MAY be proposed in patients with severe xerophthalmia. | | | 0.374 | 2 | 5.35 |
| The use of scleral lenses SHOULD be considered in patients with severe xerophthalmia and/or scarring. | | | 0.292 | 2 | 6.85 |
| Surgical ocular surface reconstruction SHOULD be considered as a last resort in patients with extensive scarring. | | | 0.724 | 3.72 | 5.14 |
| Genital area | | |  |  |  |
| Sequelae such as vulvodynia, vulvar and vaginal synechiae SHOULD be assessed after epithelialization. | | | 0.018 | 0.15 | 8.238 |
| Topical corticosteroids SHOULD be considered in patients with vulvar and/or vaginal synechiae to reduce extensive scarring. | | | 0.292 | 2 | 6.85 |
| Surgical correction SHOULD be considered in cases of extensive vulvar and/or vaginal scarring. | | | 0.132 | 1 | 7.6 |
| Emollients SHOULD be used to avoid vulvar and vaginal dryness. | | | 0.132 | 1 | 7.6 |
| Mental health | | |  |  |  |
| Every follow-up control SHOULD include a screening for psychological well-being. | | | 0.132 | 1 | 7.6 |
| This screening SHOULD include questions on the quality of sleep, mood status, anxiety, nightmares, and symptoms of depression. | | | 0 | 0 | 8.35 |
| A standardized tool such as hospital anxiety and depression score (HADS) MAY be helpful in the screening for psychological well-being. | | | 0.132 | 1 | 7.6 |
| Psychological support SHOULD be actively offered to patients with chronic disabling sequelae. | | | 0 | 0 | 8.35 |
| A psychological and/or psychiatric follow-up CAN help to reduce issues like post-traumatic stress disorder. | | | 0 | 0 | 8.35 |
| Iatrogenic psychiatric symptoms SHOULD be excluded. | | | 0.292 | 2 | 6.85 |
| Psychotropic drugs MAY be considered according to the psychiatrist’s evaluation. | | | 0.292 | 2 | 6.85 |
| Allergy workup | | |  |  |  |
| A preliminary allergy card prohibiting the use of ALL suspect drugs MUST be given to the patient upon release from the hospital. | | | 0 | 0 | 8.35 |
| The patient MUST be clearly informed during the hospital stay about the suspect drug(s), their avoidance and cross-reactivity. | | | 0 | 0 | 8.35 |
| The patient’s companion/family MUST be clearly informed during the hospital stay about the suspect drug(s), their avoidance and cross-reactivity. | | | 0 | 0 | 8.35 |
| Prick tests SHOULD NOT be routinely performed. | | | 0.132 | 1 | 7.6 |
| Intradermal tests SHOULD NOT be routinely performed. | | | 0.292 | 2 | 6.85 |
| If available, a lymphocytic transformation test (LTT) CAN be useful in the diagnostic work-up. | | | 0.492 | 3 | 6.1 |
| If available, an Elispot test CAN be useful in the diagnostic work-up. | | | 0.748 | 4 | 5.35 |
| A drug CANNOT be excluded as culprit agent solely based on negative results of any of the allergological tests. | | | 0 | 0 | 8.35 |
| A definitive allergy card MUST be given to the patient after the allergy work-up. | | | 0 | 0 | 8.35 |
| The patient MUST be clearly informed about the drug(s) on the allergy card, their avoidance and cross-reactivity after the allergy work-up. | | | 0 | 0 | 8.35 |
| The general practitioner and all physicians involved in the management of the patient MUST be informed about the drug(s) on the allergy card, their avoidance and cross-reactivity after the allergy work-up. | | | 0 | 0 | 8.35 |
|  |  |  |  |  |  |
| Eyes | | |  |  |  |
| Corneal transplantation SHOULD NOT be recommended due to the risk of clinical exacerbation. | | | 0.652 | 3 | 4.6 |
| Mental health | | |  |  |  |
| Additional measures such as hypnosis MAY help reducing symptoms of anxiety or depression. | | | 0.652 | 3 | 4.6 |
| Allergy workup | | |  |  |  |
| Allergological testing SHOULD be performed at least 6-8 weeks after complete re-epithelization. | | | 0.652 | 3 | 4.6 |
| Patch-tests SHOULD be performed for the diagnostic work-up. | | | 0.519 | 2 | 3.85 |

* *A disagreement index value greater than 1 indicates a lack of consensus; below 1 indicates a consensus.*
